# Sleep bruxism: a novel theoretical perspective through the lens of the free energy principle (FEP)

**DOI:** 10.3389/fnbeh.2026.1920406

**Published:** 2026-08-14

**Authors:** Edward Owens, Pedro Mayoral

**Affiliations:** Beacon Consultants Sleep Health Clinic, Dublin, Ireland

**Keywords:** active inference, free energy principle, phenotypes/endophenotypes, predictive processing, rhythmic masticatory muscle activity, sleep bruxism

## Abstract

Sleep bruxism is a prevalent sleep-related motor phenomenon characterized by rhythmic masticatory muscle activity that ranges from physiological behavior to a condition with clinically significant craniofacial consequences. Existing models emphasize multifactorial risk factors and centrally mediated dysregulation but lack a unified mechanistic account linking neural regulation, autonomic physiology, airway function, craniofacial biomechanics, and the temporal organization of rhythmic masticatory muscle activity. In this Hypothesis and Theory article, we outline a Free Energy Principle (FEP) – informed framework in which sleep bruxism is interpreted as an expression of predictive regulation and active inference within nested craniofacial, autonomic, and respiratory systems, explicitly as a theoretical account rather than as an empirically established mechanism. Within this framework, we introduce the construct of homeostatic latency, defined conceptually as the temporally extended window over which polysomnographic and electromyographic indices of sleep bruxism frequency, intensity, and inter-episode intervals reflect the efficiency of recovering a quasi-stable equilibrium after arousal-related perturbations. From this, we derive testable hypotheses regarding: (i) clustering of rhythmic masticatory muscle activity around micro-arousals and respiratory events, (ii) characteristic autonomic signatures preceding episodes, (iii) homeostatic latency profiles derived from EMG timing measures, (iv) airway-related phenotypes and responses to mandibular advancement devices, and (v) differentiated profiles across stress-related, arousal-related, respiratory – airway protective, and neuromodulatory sleep bruxism endotypes. In addition, we briefly indicate how an FEP-informed ontological framework for SB could, in future work, be extended into a domain-specific, knowledge-graph – based representation to support integration of multimodal data and hypothesis-driven computational modeling, while emphasizing that such tools are not developed or validated in the present article. Overall, the framework aims to reconcile sleep bruxism as both behavior and condition, in line with contemporary consensus views, and to generate falsifiable predictions for future empirical studies.

## 1 Introduction

**FIGURE 1:**
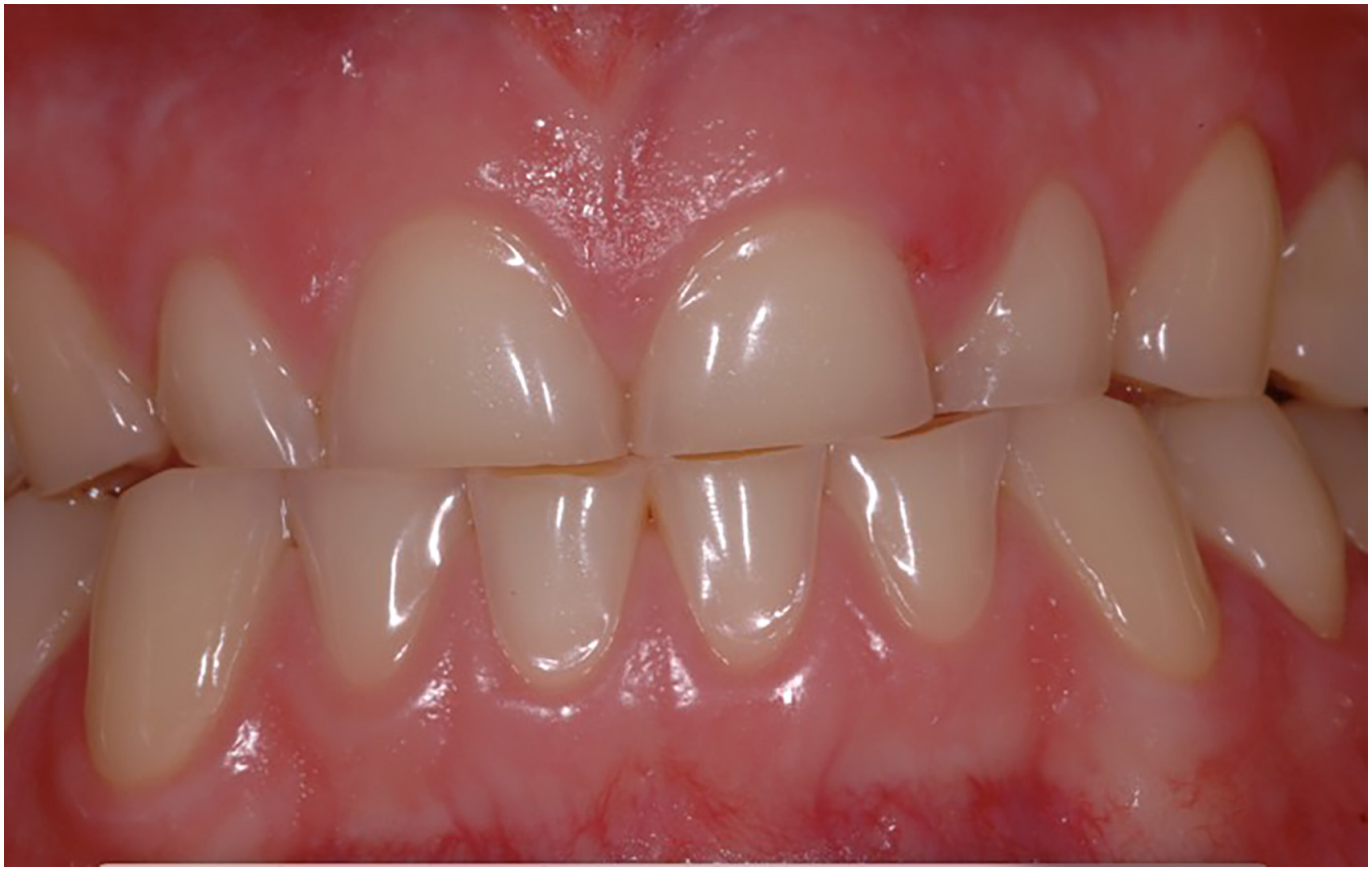
Clinical consequences of sleep bruxism. Representative intraoral images illustrating commonly reported dental and oral consequences associated with sleep bruxism, including tooth wear facets, fissures, cracks or fractures, restorative material damage, mandibular tori, tongue and cheek indentations, and masticatory muscle hypertrophy.

Sleep bruxism (SB) has been historically defined as an unconscious, neurologically mediated rhythmic masticatory muscle activity (RMMA) disorder (Carra et al., 2012; Lavigne et al., 2021; Lobbezoo et al., 2013). SB can present with rhythmic, clonic, and mixed patterns of varying frequency, intensity and duration (Mayer et al., 2016). ICD-11 classifies SB as a sleep-related movement disorder and further distinguishes neurological and psychogenic origins as different categories of SB (World Health Organization, 2019). Clinically, SB is characterized by excessive, involuntary jaw thrusting, clenching and/or teeth grinding (Carra et al., 2012; Lobbezoo and Naeije, 2001; Lobbezoo et al., 2018; Meira e Cruz, 2026). The global prevalence of SB is estimated at approximately 19–30% of the population, depending on diagnostic criteria and assessment methods (Melo et al., 2019). SB can lead to dental complications such as tooth wear, fissures, cracks or fractures, and tooth mobility (Beddis et al., 2018; Figure 1), and may also affect oral and craniofacial tissues, presenting with mandibular tori, tongue and cheek indentations, masticatory muscle hypertrophy, temporomandibular joint pain or locking, headaches, and masticatory muscle fatigue or pain (Palinkas et al., 2015). In prosthodontically restored dentitions, damage to restorative materials is a common finding, and SB is reported as a risk factor for complications in dental implant therapy (Ionfrida et al., 2025).

The diagnosis of SB can be approached through a combination of patient-reported symptoms, clinical signs, and standardized questionnaires, supplemented by instrumental assessments such as SB indices, digital STL difference files, and intraoral sensors (Manfredini et al., 2022; van Nistelrooij et al., 2024; Cid-Verdejo et al., 2024; Li et al., 2024). The definitive instrumental assessment of SB is achieved through polysomnography (PSG) with audio–video recording, including sleep staging, EMG assessment of masticatory muscles, detection of sleep arousals with associated RMMAs, and heart rate variability analysis (Stuginski-Barbosa et al., 2017; Miettinen et al., 2020). SB events are typically classified as low frequency when recorded two to four times per hour of sleep and as mild to high frequency when occurring more than four up to 25 times per hour, with an EMG amplitude of at least 10% of maximum masseter clenching activity while awake (Kato et al., 2001; Lavigne et al., 2001; Rompré et al., 2007). These criteria, together with clinical, instrument-based, and patient-reported measures, provide a multifaceted characterization of SB.

SB is currently recognized as a multifactorial phenomenon, with evolving concepts framing it both as a behavior that may be within physiological limits for many individuals and, in other cases, as a parafunctional disorder with pathophysiological consequences in the craniofacial complex (Kim and Shin, 2024; Michelotti et al., 2010; Cameron, 1964). Contemporary consensus suggests that SB is best understood as a centrally regulated, context-dependent motor activity, which can be adaptive or maladaptive depending on its intensity, frequency, and clinical sequelae, and is primarily associated with central rather than purely peripheral regulation (Uchima Koecklin et al., 2024; Lavigne et al., 2007). This central weighting is believed to be driven by dysregulated sensorimotor integration, altered brainstem and cortical excitability, and fluctuations in aminergic autonomic, nociceptive, and proprioceptive neural circuitry (Uchima Koecklin et al., 2024; Lavigne et al., 2007). Exposure to chronic stress and medication-associated expressions are considered significant contributors to SB presentations (Pavlou, 2024; Garrett and Hawley, 2018; Winocur et al., 2003), and SB is also highly prevalent in adults with obstructive sleep apnea (OSA) (Li et al., 2023; Sambale et al., 2025; Smardz et al., 2022).

Within this framework, SB reflects the dynamic interplay of autonomic activation, cortical arousals, respiratory disturbances, and comorbid conditions that destabilize sleep physiology, while premature contacts and occlusal interferences, though not regarded as primary etiological factors, are considered peripheral mechanical triggers that may modulate its occurrence and amplify its clinical consequences (Lavigne et al., 2007; Kato et al., 2001; Mayer et al., 2016; Souza et al., 2020; Thomas et al., 2024). This centrally mediated, multifactorial model naturally invites mechanistic accounts grounded in brain–body regulation and predictive control (Friston, 2010). In particular, it motivates theoretical perspectives that can jointly address neural regulation, autonomic physiology, airway function, craniofacial biomechanics, and the temporal organization of RMMA within a single framework.

The Free Energy Principle (FEP), formulated by Karl Friston, is one such theoretical framework, proposing that living systems act to minimize a quantity called variational free energy (VFE), which bounds informational surprise or uncertainty and thereby supports the maintenance of their characteristic states and organization (Friston, 2012). In biological applications, the FEP has been advanced as a candidate unifying theory of brain function, positing that organisms regulate and minimize VFE to sustain homeostasis and can also implement adaptive allostatic behaviors, effectively reducing expected surprise about their sensory and physiological states (Ororbia and Friston, 2023; Tschantz et al., 2022). The brain is conceived as continuously constructing and updating internal generative models to predict sensory inputs (predictive processing) and reducing discrepancies – prediction or model errors – through perceptual inference (belief updating) and active inference (action) (Parr and Friston, 2018).

In its broadest formulation, this process is proposed to govern neurological, physiological, and behavioral regulation, enabling organisms to optimize survival by minimizing uncertainty or surprise in their environment (Conant, 1970; Friston, 2010; Parr et al., 2022). The FEP is increasingly being used to explore maladaptive pathological processes, including aberrant motor patterns such as tremors and dyskinesias (Badcock et al., 2022; Münchau et al., 2024). The theoretical conjecture is that, in such pathological presentations, inaccurate prediction and control of movement that fail to effectively minimize informational “free energy” may be associated with increased thermodynamic energy dissipation or entropy (Kempster and Perju-Dumbrava, 2021). At the same time, there is ongoing discussion about the scope and limitations of FEP-based models in biological and cognitive science, and their application to specific physiological systems should be regarded as hypothesis-generating rather than definitive.

The FEP describes how living systems preserve their structure by continually minimizing VFE, a process that is mathematically related to Bayesian inference (Kim, 2023; Friston, 2012). This allows these systems to update their beliefs (priors) and reduce uncertainty about hidden causes in their environment (Friston, 2009). Under this view, VFE minimization is achieved by constructing and refining internal generative models that predict sensory inputs, with the system acting to resolve discrepancies between predictions and observed data (Tschantz et al., 2022). Central to this framework is the concept of the Markov blanket (MB), which statistically delineates the boundary or interface between a system’s internal states and its external environment, mediating their interaction via sensory and active states (Kirchhoff et al., 2018; Parr and Friston, 2019). This formalism enables the partitioning of complex systems and supports the notion of nested Markov blankets: hierarchically organized sub-systems (e.g., cells, organs, organisms) each possess their own MB, allowing for multi-level autonomy and self-organization (Kirchhoff et al., 2018).

Active inference is the process theory derived from the FEP, describing how biological agents use a generative model to predict sensory inputs and select actions that minimize expected free energy, thereby maintaining homeostasis and preferred states (Friston, 2022; Zhang and Xu, 2024; Parr and Friston, 2019). This bi-directional dynamic process inherently relies on Bayesian inference, as agents update their beliefs/priors about hidden states of the world based on sensory evidence, optimizing perception and action through probabilistic reasoning and precision weighting. High precision on interoceptive error drives motor responses, while low precision favors model revision (Gottwald and Braun, 2020; Isomura, 2022; Tschantz et al., 2022). Together, these components provide a unified account of perception, learning, and action in biological systems, grounded in the minimization of VFE and the statistical structure imposed by an MB (Zhang and Xu, 2024; Parr and Friston, 2019; Ramstead et al., 2021).

Considering SB as a multifactorial phenomenon with a strong neurological weighting in its manifestation as both a behavior and, in some individuals, a disorder, the FEP offers a candidate mechanistic framework for interpreting its varied observable clinical phenotype presentations. Within this perspective, a pathophysiological SB presentation can be conceptualized as a potentially aberrant or dysfunctional expression of predictive regulation and active inference in the craniofacial, orofacial sensorimotor, and autonomic domains (Friston, 2010; Kim, 2023). Importantly, in the present article these accounts are advanced as theoretical interpretations and sources of testable hypotheses, rather than as mechanisms already demonstrated empirically in SB.

The aim of this paper is therefore to develop a more precise theoretical model of SB that aligns with the principles of the FEP, achieved through a comprehensive, inclusive ontological framework describing its putative underlying mechanisms. The proposed model is intended to (i) organize existing observations into a coherent endotype-based account, (ii) generate falsifiable predictions for polysomnographic, electromyographic, autonomic and clinical indices, and (iii) suggest how future work might refine descriptive grading and diagnostic criteria that are currently fragmented across neurology, dentistry and sleep medicine (Wu et al., 2025). In addition, we outline how this framework could eventually inform more predictive, individualized, and targeted management strategies for SB, while emphasizing that such clinical applications remain prospective and require substantial empirical validation.

## 2 Specific integration of free energy principle within a sleep bruxism ontology

This section develops a conceptual ontological framework for SB grounded in the FEP, using Markov blankets and free-energy minimization as organizing tools to describe its neural, biomechanical, and temporal organization. Within this perspective, SB motor behaviors are interpreted as potential expressions of action-perception cycles that arise in response to multimodal sensory inputs (interoceptive, proprioceptive, nociceptive), with these cycles hypothetically acting to minimize discrepancy or “surprise” between internal generative models and received sensory inputs (Tschantz et al., 2022; Adams et al., 2013). These accounts are advanced as theoretical interpretations that extend existing empirical observations on RMMA timing and autonomic–cortical activation, rather than as mechanisms already demonstrated in SB.

### 2.1 Key elements of the free energy principle application in SB

#### 2.1.1 Markov blanket (MB)

**FIGURE 2:**
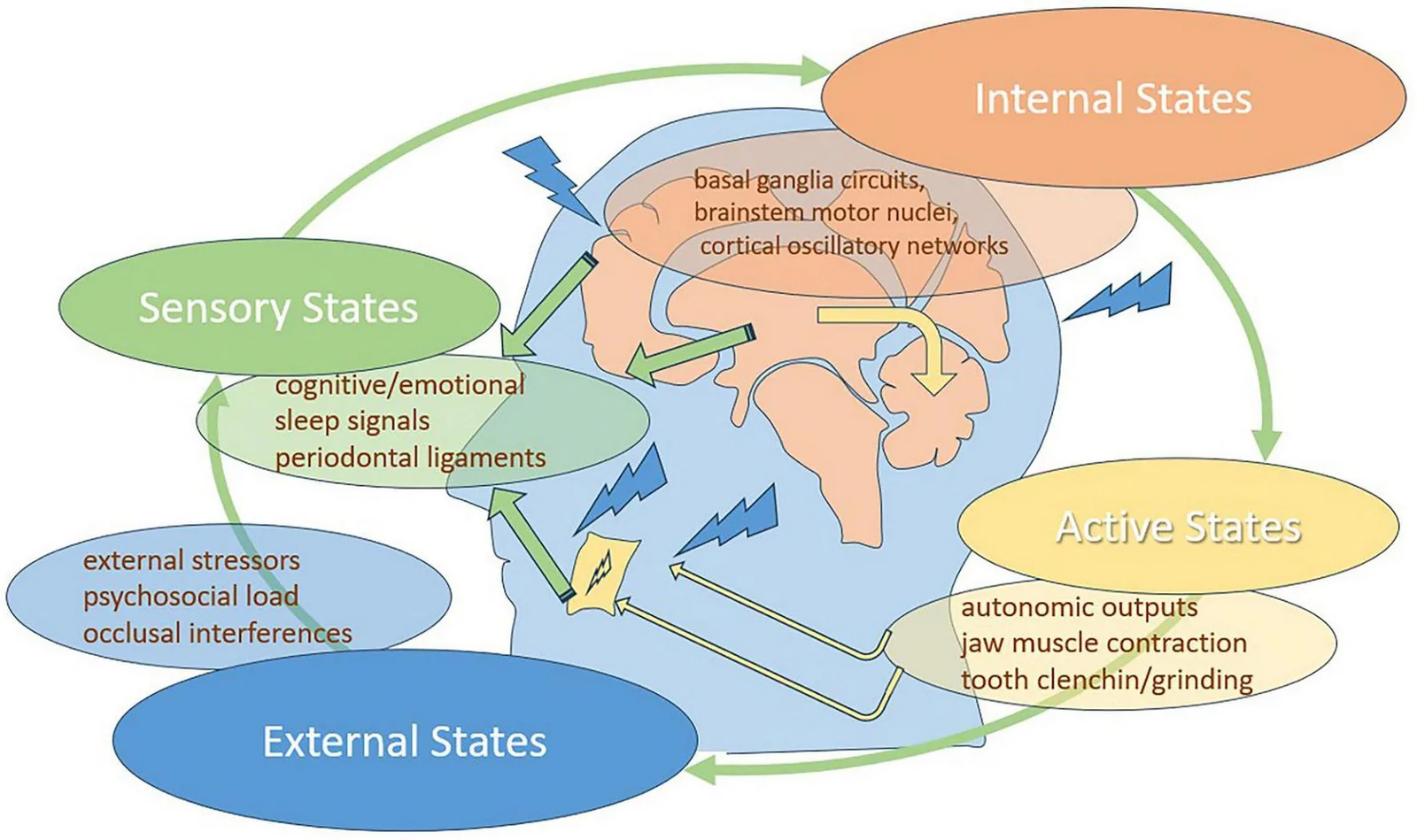
Sleep bruxism modeled as a Markov blanket. Schematic representation of sleep bruxism within a Free Energy Principle–informed framework, showing internal states, external states, and the sensory and active states that mediate their interaction. The figure is intended as a conceptual partition for organizing putative information flow in sleep bruxism, rather than as a literal anatomical map or a directly validated mechanistic model.

From the perspective of the FEP, SB can be modeled conceptually as a system with a Markov blanket (MB) that statistically separates the internal states of the organism from its external environment (Kirchhoff et al., 2018; Parr et al., 2020; Virgo et al., 2022; Bruineberg et al., 2022). In this formalism, the MB consists of sensory and active states that mediate the interaction between internal and external states through a generative model (Figure 2).

In the context of SB, the internal states correspond to central neural substrates and physiological processes that generate predictions about orofacial system stability; these include basal ganglia circuits, brainstem motor nuclei, and cortical oscillatory networks involved in motor control and sleep regulation (Thomas et al., 2024; Giovanni and Giorgia, 2021). The external states include biomechanical and environmental factors outside direct neural control, encompassing external stressors such as noise and psychosocial load, as well as biomechanical constraints including tooth alignment, occlusal interferences, and mandibular anatomy (Giovanni and Giorgia, 2021; Uchima Koecklin et al., 2024). These external states are not directly represented in the nervous system but influence the sensory input that reaches it (Mayer et al., 2016; Lavigne et al., 2008; Thomas et al., 2024; Kostrzewa-Janicka et al., 2015).

The sensory states are shaped by afferent signals reaching the nervous system. They arise both from central neuroregulatory processes (cognitive-emotional modulation, sleep-wake regulation, autonomic balance) and from peripheral sensory feedback provided by periodontal ligaments, temporomandibular joint receptors, and orofacial muscles, which together inform the brain of discrepancies between predicted and actual jaw-tooth contact (Giovanni and Giorgia, 2021; Lavigne et al., 2001). These states encode the consequences of actions and environmental changes. The active states correspond to efferent motor outputs that act on the environment. In SB, these include motor commands driving jaw muscle contractions, jaw thrusting, tooth clenching or grinding, as well as autonomic outputs that modulate cardiac and respiratory activity during SB episodes (Mayer et al., 2016; Giovanni and Giorgia, 2021; Lavigne et al., 2007). In the present work, this MB-based description is used as an abstract modeling device to structure hypotheses about SB regulation, not as a direct anatomical identification of MB *in vivo*.

#### 2.1.2 Nested MB

The FEP framework supports hierarchical organization through nested Markov blankets, used here as a way to describe multi-level structure in the systems involved in SB. These conceptual partitions delineate information flow across scales of neural and motor organization. Craniofacial and neuromuscular systems, when represented as nested MBs, can be used to describe top-down and bottom-up information flow between physiological states and behavioral outputs – for example, a trigeminal–jaw sensorimotor “blanket” nested within autonomic–arousal and respiratory–airway “blankets” governing sleep-breathing stability.

At the lowest level, individual neurons and local circuits – such as mesencephalic trigeminal nucleus neurons – can be idealized as MBs separating their internal states from synaptic and sensory inputs, mediating the flow of information between jaw muscle stretch receptors and central processing units (Giovanni and Giorgia, 2021; Hipólito et al., 2021). At the next level, brainstem nuclei such as the ascending reticular activating system act as functional units with their own MBs, integrating signals from the mesencephalic trigeminal nucleus and modulating arousal, autonomic, and motor outputs (Giovanni and Giorgia, 2021; Lavigne et al., 2007). Cortical regions involved in motor planning and sleep regulation form higher-order MBs, coordinating with subcortical and muscular systems during sleep stage transitions and cortical micro-arousals (Zhu et al., 2024). Finally, the masticatory muscles themselves, as effectors, can be treated as bounded by MBs that mediate the interface between neural commands and biomechanical activity, particularly during RMMA episodes in SB (Giovanni and Giorgia, 2021; Lavigne et al., 2007).

These nested MBs, understood as modeling constructs, enable a description of multi-level autonomy and conditional independence between subsystems, thereby providing a structured way to think about coordinated generation of RMMA and associated autonomic changes during SB (Hipólito et al., 2021; Palacios et al., 2020). We use this language as a scaffold for the ontology, without implying that MB boundaries are directly observable anatomical entities.

#### 2.1.3 Active inference and Bayesian inference

Active inference and Bayesian inference are central to the application of the FEP in understanding SB, as they offer a process-level account of how the brain might predict, interpret, and respond to internal and external sensory signals during sleep. Under the FEP, the brain is conceptualized as a Bayesian inference engine that constructs and continuously updates a generative model to minimize prediction error and uncertainty about bodily states and environmental cues, including those related to masticatory muscle activity and arousal events in SB (Zhang and Xu, 2024; Isomura, 2022; Kim, 2021, 2023).

Active inference extends this framework by proposing that the brain not only passively perceives but also actively selects motor actions (policies) – such as rhythmic jaw movements – based on predictions that minimize expected free energy and thereby help maintain physiological homeostasis and preferred states during sleep (Zhang and Xu, 2024; Kim, 2021, 2023). This process involves the integration of sensory feedback, such as proprioceptive and nociceptive signals from jaw muscles, with top-down predictions from cortical and brainstem networks, with the MB formalism delineating the boundaries between neural and muscular subsystems (Isomura, 2022; Ramstead et al., 2021).

In SB, aberrant or excessive motor activity is hypothesized to reflect maladaptive inference or imprecise prediction-error weighting within these nested MBs, leading to dysregulated motor output and state transitions. In particular, chronic alterations in the “precision” assigned to proprioceptive and nociceptive bottom-up sensory input may, in principle, bias the system toward habitual, high-gain bruxism responses. In the present article, this formalism is used strictly as a theoretical model of how motor output and sensory feedback could be coordinated in SB, rather than as a directly measured quantity (Kempster and Perju-Dumbrava, 2021; Kim, 2023). Thus, the FEP, through active and Bayesian inference, provides a unified computational framework for generating hypotheses about the perception–action cycle and its dysregulation in conditions such as SB.

#### 2.1.4 Kulllback–Leibler divergence (KL)

Kullback–Leibler (KL) divergence is the mathematical backbone of the FEP in explaining perception, action, and learning, as it quantifies the difference between internal probabilistic beliefs or priors and the true distribution of external states, thereby driving the minimization of VFE (Parr and Friston, 2019; Zhang and Xu, 2024; Mazzaglia et al., 2022). Minimizing VFE is mathematically equivalent to minimizing the KL divergence between the variational density – the internal state’s beliefs about external states given sensory input – and the true posterior distribution of external states given sensory input (Parr and Friston, 2019; Zhang and Xu, 2024; Mazzaglia et al., 2022).

This minimization process underlies perception through belief updating, action through selection of outputs that minimize expected free energy, and learning through refinement of the generative model (Parr and Friston, 2019; Zhang and Xu, 2024; Mazzaglia et al., 2022). The MB formalism provides the structural basis for this process by partitioning the system, establishing conditional independence and regulating information flow between the system and its environment (Parr and Friston, 2019; Ramstead et al., 2021; Raja et al., 2021). In the context of SB, KL divergence is used here as a formal measure of the mismatch between the brain’s predictions and actual sensory states, with bruxism conceptualized as a possible motor strategy to reduce this divergence. This remains a theoretical construct rather than an empirically quantified value in current SB research.

#### 2.1.5 Biorhythms

As a multiscale nested system of MBs, the craniofacial system can be described as existing in a non-equilibrium steady state (NESS) characterized by biorhythms. Biorhythms are recurrent physiological cycles, such as heart-rate variability and sleep stage transitions, that reflect the dynamic balance between regulatory (conservative) and adaptive (dissipative) processes in NESS systems (Shiraishi et al., 2021; Lavigne et al., 2007; Palacios et al., 2020). In SB, these biorhythms are tightly linked to the timing and occurrence of RMMA, with episodes often preceded by transient increases in autonomic and cortical activity, including heart-rate accelerations and micro-arousals (Shiraishi et al., 2021; Lavigne et al., 2007; Kato et al., 2001).

Within the FEP framework, biorhythms are interpreted as reflecting the system’s tendency to return to an attracting set of states, minimizing prediction error and maintaining homeostasis around a specific attractor or set point in response to internal and external perturbations (Bettinger and Friston, 2023). This NESS dynamical-systems conceptualization is consistent with the observed rhythmic and clustered patterns of SB across sleep cycles, and with the impact of disrupted sleep architecture or biological rhythm – such as irregular sleep patterns, insomnia, or altered autonomic–cardiac cycles – on SB frequency and severity (Zhu et al., 2024). Thus, in our ontology, biorhythms are treated as integral to applying the FEP in SB, as they mediate the interplay between autonomic, neural, and muscular systems in the maintenance of physiological stability.

This entails that living systems essentially maintain a NESS, a characteristic probability distribution over their states that defines what an organism is, with the density of this distribution corresponding to the clinically observable organism phenotype (Ramstead et al., 2021; Tschantz et al., 2022).

### 2.2 Integrative framework of homeostatic, homeorhetic, and allostatic mechanisms in sleep bruxism

**FIGURE 3:**
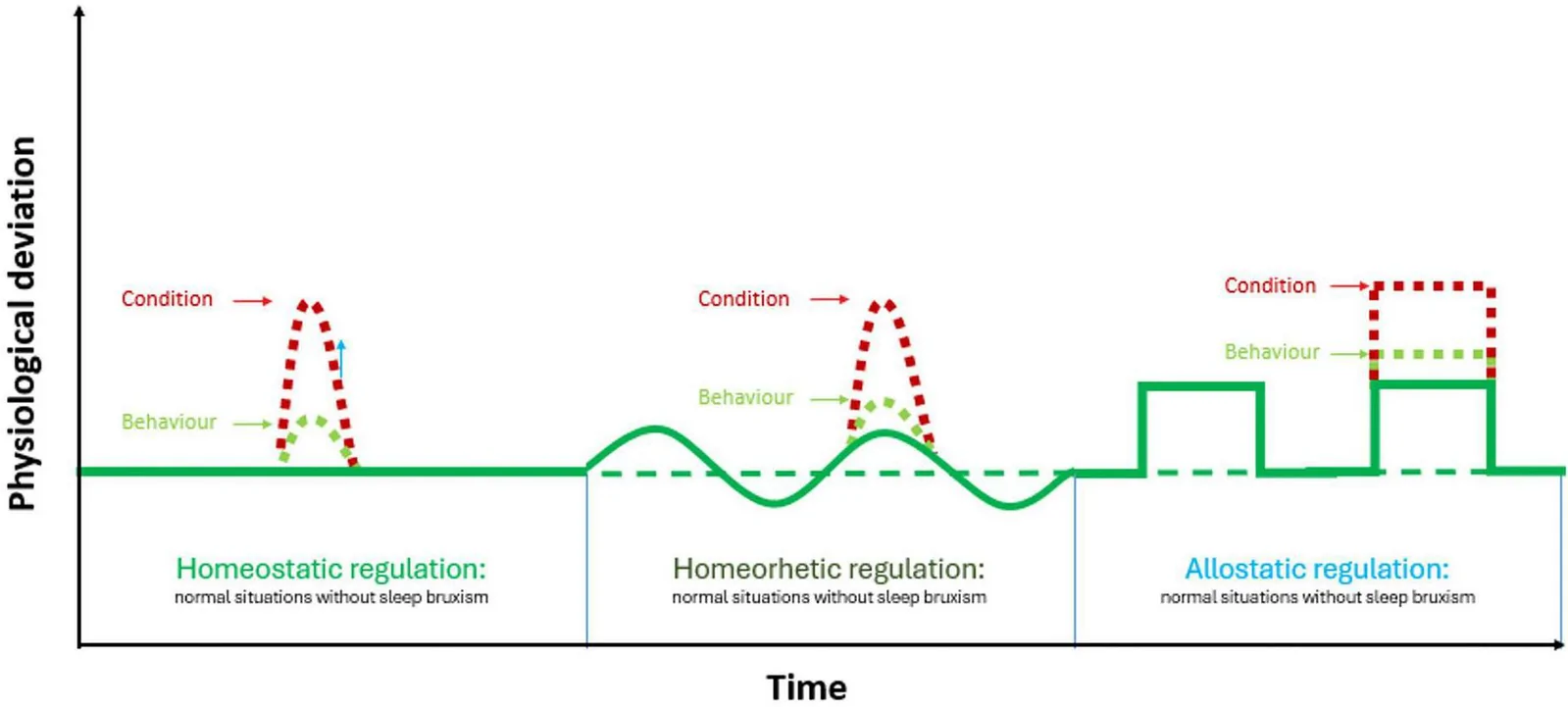
Homeostatic, homeorhetic, and allostatic regulation of sleep bruxism. Conceptual diagram illustrating how short-term homeostatic regulation, trajectory-level homeorhetic organization, and anticipatory allostatic regulation may contribute to the temporal patterning of rhythmic masticatory muscle activity during sleep. Within the present theoretical framework, these regulatory modes are used to organize sleep bruxism along a continuum from contextually adaptive behavior to higher-cost, potentially maladaptive expressions.

An integrative framework encompassing homeostatic, homeorhetic, and allostatic mechanisms provides a neurophysiologically coherent conceptual model for understanding the regulation of SB (Figure 3). The core idea is that biological systems minimize VFE to maintain internal stability and adapt to changing demands (Friston, 2012). Within this view, homeostatic regulation, homeorhetic trajectories, and predictive allostatic behaviors can be interpreted through the nested MB construct (Ororbia and Friston, 2023). Collectively, these regulatory modes are posited to contribute to the stability, adaptability, and resilience of motor and autonomic systems in both physiological and pathological contexts (Cano-Martínez et al., 2024; Stevenson, 2024; Bettinger and Friston, 2023).

**Homeostatic regulation.** Homeostatic mechanisms act over short timescales to maintain immediate physiological stability. In normal individuals, these mechanisms regulate jaw muscle tone and autonomic activity across sleep-wake states, ensuring balanced muscle relaxation and preventing fatigue (Toossi et al., 2017). In SB, homeostatic control manifests as rapid, transient adjustments of jaw muscle activity and sympathetic responses during micro-arousals (Lavigne et al., 2007). RMMA episodes often coincide with brief increases in cortical and autonomically mediated cardiac activation, helping to restore airway patency and stabilize internal variables such as oxygenation and muscle tone within narrow ranges (Miettinen et al., 2020). These events can be interpreted as examples of moment-to-moment equilibrium maintenance via continuous minimization of sensory-motor prediction errors.

**Homeorhetic regulation.** Homeorhetic mechanisms reflect the dynamic stability of physiological systems, emphasizing maintenance of stable functional trajectories rather than fixed states. In healthy individuals, rhythmic masticatory muscle activity follows consistent low-frequency, low-amplitude patterns that adapt across sleep stages, reflecting an organized relationship with the sleep cycle and transitions between non-REM and REM states (Zhu et al., 2024; Toyota et al., 2022). In SB, this homeorhetic organization may persist despite perturbations, maintaining functional continuity across cycles of varying arousal and autonomic tone (Shiraishi et al., 2021). RMMA episodes tend to cluster during lighter N1 and N2 sleep stages and the ascending phase of sleep cycles, suggesting that the system sustains a trajectory that supports recovery and adaptation without reverting to rigidity or collapse (Toyota et al., 2022; Kishi et al., 2020). This dynamic balance – flexibility within stability – is characteristic of self-organizing biological systems operating under free-energy minimization.

**Allostatic regulation.** Allostatic mechanisms extend these principles by enabling predictive and anticipatory adjustments in physiological systems. Under normal conditions, masticatory and autonomic networks proactively modulate muscle tone and arousal thresholds in expectation of changing internal or external demands, such as stress or sleep fragmentation (Kishi et al., 2020; Shiraishi et al., 2021). In SB, predictive allostasis becomes evident when neural and autonomic circuits adjust to anticipated challenges – such as transient airway narrowing or psychosocial stress – by increasing RMMA frequency or muscle readiness (Shiraishi et al., 2021). These anticipatory behaviors may reduce potential mismatches between expected and actual sensory feedback, thereby minimizing prediction error and preserving systemic integrity (Shiraishi et al., 2021; Zhong et al., 2020; Pavlou, 2024). When this adaptive allostatic capacity is exceeded or dysregulated, maladaptive bruxism state-transition patterns may emerge, illustrating how predictive regulation might bridge physiological stability and pathology within the FEP framework (Kempster and Perju-Dumbrava, 2021).

### 2.3 Homeostatic latency and predictive regulation in sleep bruxism

During sleep, time-dependent neurophysiological processes restore functional stability within masticatory and central motor control networks between successive RMMA episodes. These processes, encompassing both homeostatic and arousal-related mechanisms, down-regulate motor excitability following bursts of muscular activation and transient arousal events. Consequently, parameters such as inter-episode intervals, RMMA frequency, and contraction intensity may be regarded as candidate indices of underlying homeostatic control governing motor output and adaptive recovery across the sleep period.

**FIGURE 4:**
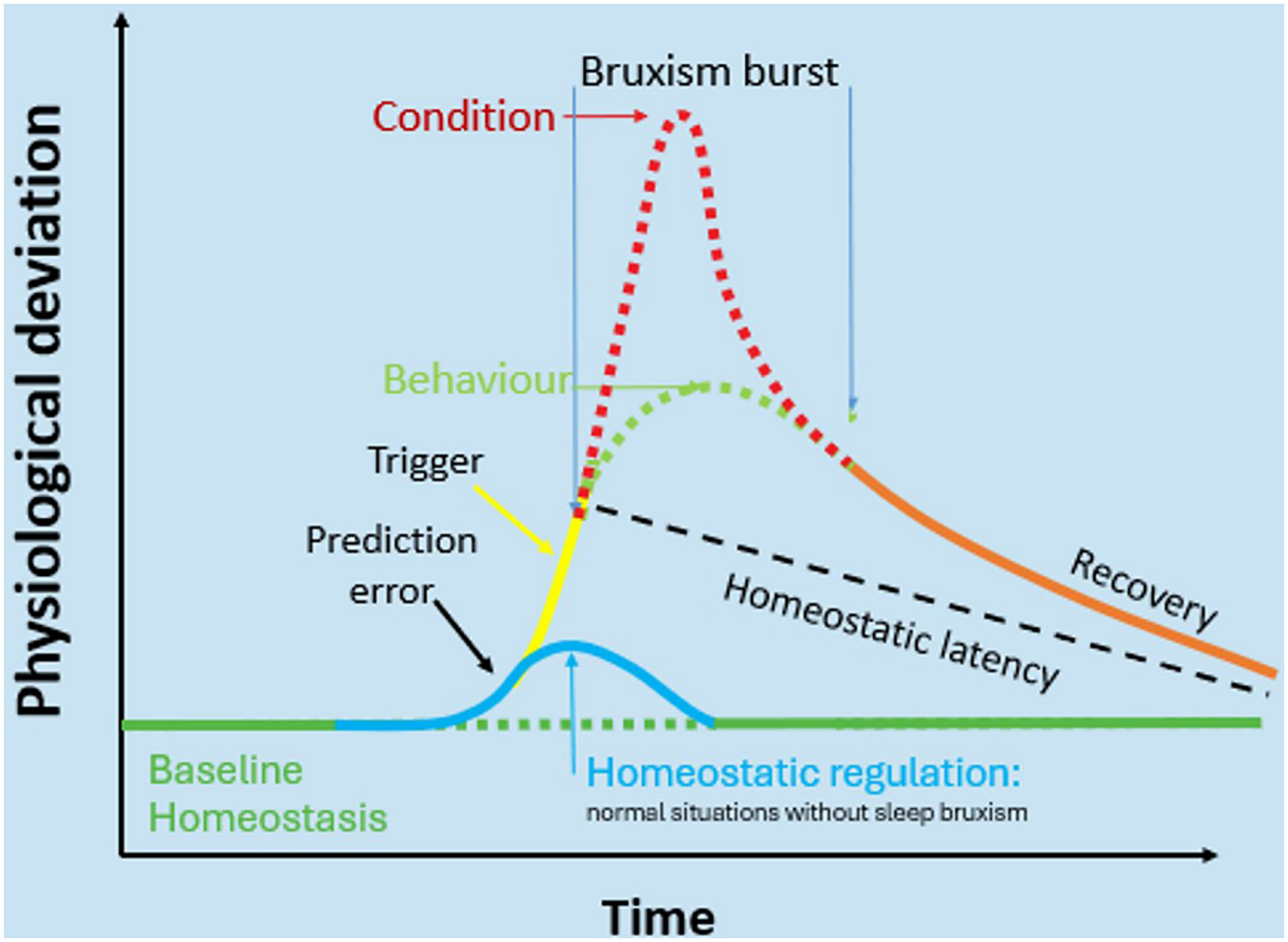
Homeostatic latency as a temporal metric of sleep bruxism regulation. Conceptual illustration of homeostatic latency, defined here as a hypothesized temporal window linking a perturbation such as micro-arousal, airway-related disturbance, or stress-related arousal to a return toward quasi-stable physiological organization. The figure summarizes the proposed relation between event frequency, inter-episode interval, and intensity, and is intended to present a testable construct rather than an established clinical metric.

In individuals across the spectrum of SB clinically observable phenotypes, RMMA follows a periodic pattern, with event frequency, duration, and inter-episode spacing reflecting individual profiles of adaptive motor regulation (Lavigne et al., 2007; Kato et al., 2001). To capture the temporal organization of these regulatory processes, we introduce the construct of homeostatic latency, defined as the composite temporal window encompassing the frequency, interval, and intensity of SB events (Figure 4). Conceptually, this parameter represents the delay between a transient physiological perturbation (e.g., airway obstruction, micro-arousal, or emotional stress) and the attainment of a quasi-stable equilibrium in arousal and motor activity, delineating the temporal threshold at which regulatory mechanisms re-establish balance following an episode of motor activation.

From the perspective of the FEP, the temporal dynamics of RMMA and SB may be interpreted as expressions of transient mismatches between endotype-specific generative models of bodily states and incoming interoceptive or proprioceptive feedback (Zhu et al., 2024). Persistent prediction error over successive cycles may elicit compensatory masticatory activity aimed at restoring predictive alignment and physiological stability. In this framework, homeostatic latency is proposed as a temporal descriptor of the interval over which prediction error is either resolved or propagated, and thus as a potential indicator of whether motor responses remain adaptive or evolve into maladaptive SB patterns associated with elevated allostatic load.

Under typical, non-bruxism conditions, homeostatic regulation ensures rapid stabilization of jaw muscle tone during sleep, maintaining low baseline electromyographic (EMG) activity and preventing excessive contractions (Lavigne et al., 2001). Homeorhetic mechanisms facilitate gradual adjustment of masticatory muscle activity across sleep stages, while allostatic processes enact anticipatory modulation in response to transient arousals or environmental perturbations (Lavigne et al., 2007; Kato et al., 2001). Within this integrated model, homeostatic latency represents a conceptual convergence point of these regulatory processes – where predictive, homeorhetic, and allostatic mechanisms jointly constrain motor output to maintain equilibrium and, when dysregulated, may contribute to the transition toward repetitive, high-intensity bruxism events. At present, homeostatic latency should therefore be regarded as a hypothesis-generating construct that motivates future empirical work, rather than as an established clinical metric.

## 3 Sleep bruxism and masticatory muscle activation under the NESS framework

Within the FEP, SB can be conceptually framed as an if-then mapping from endotype to NESS: IF a specific configuration of the generative model (endotype) is present, THEN a characteristic NESS density is instantiated whose high-probability states manifest as a multidomain phenotype across structural, neurological, autonomic, neurocognitive/behavioral, and orofacial/dental domains (Uchima Koecklin et al., 2024; de Miranda Diniz et al., 2024; Lavigne et al., 2008). In this view, the phenotype of SB is not just a list of isolated symptoms, but can be interpreted as the NESS projected onto observable manifolds within the autonomic–arousal–motor (AAM) complex that characterizes a specific SB pathophysiology. This formulation is intended as a theoretical organizing device; it does not imply that such endotypes and NESS distributions have already been empirically estimated in SB.

**FIGURE 5:**
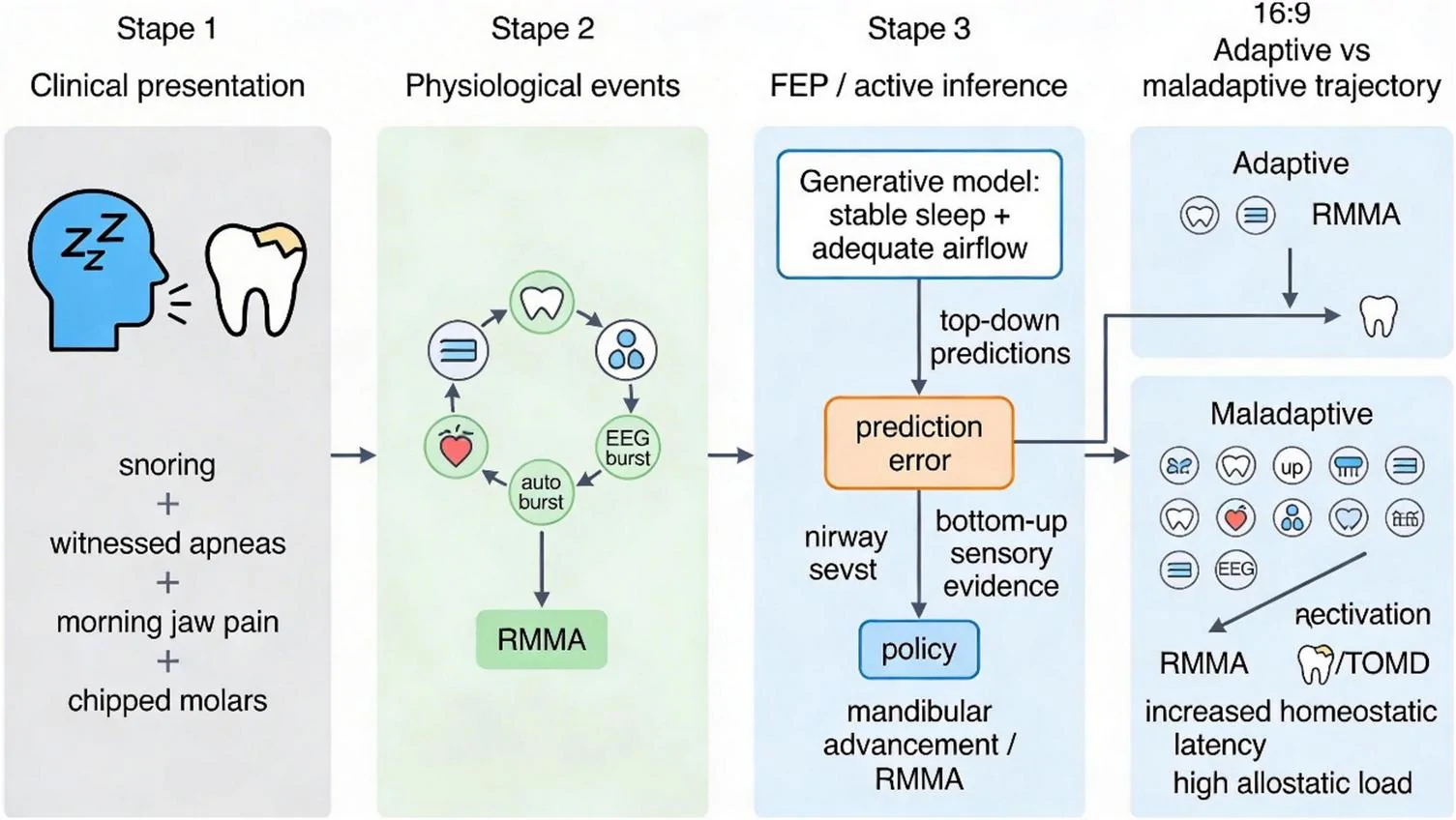
Illustrative vignette of OSA-associated sleep bruxism in an FEP-informed framework. Illustrative clinical vignette depicting a patient with obstructive sleep apnea and coexisting sleep bruxism, highlighting the empirically described sequence linking respiratory events, micro-arousals, autonomic activation, and rhythmic masticatory muscle activity, alongside the theoretical reinterpretation of this pattern within an FEP-informed model. The vignette is intended to demonstrate how the framework may be applied conceptually to a comorbid presentation, and does not constitute a validated mechanistic or decision-support pathway.

Polysomnographic evidence consistently shows that rhythmic or sustained jaw activity is preceded by sympathetic bursts, cortical micro-arousals, or respiratory events (Kato et al., 2001; Lavigne et al., 2007; Macaluso et al., 1998; de Miranda Diniz et al., 2024). Within the FEP, these phenomena can be interpreted as active-inference policies: motor responses recruited to minimize prediction error when the expected state of stable sleep and breathing is contradicted by sensory evidence (Zhang and Xu, 2024; Gottwald and Braun, 2020). Within the NESS framework, each dominant mechanism that precedes RMMA – autonomic/arousal activation, respiratory disturbance, chronic stress, or neuromodulatory dysfunction – is used here to define a hypothesized SB endotype: IF a given endotype configures the generative model with particular likelihood mappings, transition dynamics, and precision weights, THEN a corresponding NESS would include a specific pattern of AAM activation and bruxism expression across observable phenotypes. The endotypes described below are therefore working descriptors that yield testable predictions, not yet validated diagnostic entities (Figure 5).

### 3.1 Endotype descriptor framework for SB

#### 3.1.1 Autonomic/arousal endotype: sympathetic activation, cortical micro arousals and SB

SB is currently understood as a secondary phenomenon linked to arousal-related processes, rather than as a primary parafunctional motor disorder (Lavigne et al., 2007; Lavigne et al., 2008). Polysomnographic studies consistently demonstrate that RMMA episodes are temporally preceded by bursts of sympathetic activation and cortical micro-arousals, in the context of stress and respiratory apneic events (Kato et al., 2001; Lavigne et al., 2007; de Miranda Diniz et al., 2024; Macaluso et al., 1998). This sequence reflects a coordinated AAM complex, in which transient increases in EEG activity and heart rate precede jaw muscle contractions (Kato et al., 2001; Lavigne et al., 2007; Macaluso et al., 1998).

Within our framework, these findings motivate an autonomic/arousal SB endotype. Hypothetically, IF the generative model encodes heightened precision on arousal transitions and sympathetic interoceptive channels, THEN the corresponding NESS would be dominated by frequent micro-arousals, sympathetic surges, and high-frequency phasic RMMA within a permissive arousal window, yielding an elevated SB episode index and NREM-dominant phasic events with morning jaw fatigue and TMJ loading as characteristic phenotypic states. This endotype suggests specific, falsifiable predictions about PSG patterns (e.g., RMMA clustering around micro-arousals and autonomic bursts), which future studies could test prospectively.

#### 3.1.2 Stress/allostatic endotype as NESS

From the perspective of the FEP, the muscular activation that characterizes SB can also be interpreted as a possible response to stress and interoceptive input, in which motor output is recruited to minimize prediction error when physiological arousal and sensory evidence indicate instability during sleep. Stress activates neuroendocrine pathways and elevates physiological arousal, as evidenced by increased cortisol and adrenocorticotropic hormone levels in individuals with SB (Karakoulaki et al., 2015; Kaya et al., 2025; Walczyńska-Dragon et al., 2025). Polysomnographic studies further show that autonomic bursts and cortical micro-arousals commonly precede RMMA (Kato et al., 2001; Lavigne et al., 2007; Lavigne et al., 2008).

Within the NESS framework, these observations motivate a stress/allostatic SB endotype. Conceptually, IF chronic psychosocial stress, anxiety, and maladaptive coping configure the generative model with persistent allostatic load and elevated precision on threat-related arousal signals, THEN a stress/allostatic NESS would be characterized by chronic sympathetic hyperactivation, elevated cortisol and catecholamines, combined awake and sleep bruxism, and a broad orofacial pain distribution with high symptom perception (Mancini et al., 2024; Lavigne et al., 2020). This interpretation is consistent with evidence that higher perceived stress correlates with increased bruxism activity and that maladaptive coping strategies further amplify this effect (Pavlou, 2024; Saczuk et al., 2019; Fluerasu et al., 2022; Przystańska et al., 2019). In AAM terms, SB can here be viewed as an adaptive motor response that may become maladaptive when persistently recruited to resolve stress-induced arousal, integrating stress, arousal, and motor output within a single regulatory complex (Uchima Koecklin et al., 2024; de Miranda Diniz et al., 2024; Giovanni and Giorgia, 2021). At present, this endotype remains a hypothesis that suggests combined autonomic, endocrine and clinical profiling as a target for future work.

#### 3.1.3 Respiratory/airway protective endotype: sleep related breathing disorders and bruxism

Sleep-related breathing disorders, particularly obstructive sleep apnea (OSA), have been strongly associated with SB, further supporting the view that this oromotor activity may form part of an integrated AAM response (Li et al., 2023; Doblado et al., 2025; González-González et al., 2023; Martynowicz et al., 2019). Clinical and polysomnographic studies show that RMMA frequently co-occurs with respiratory events, with bruxism episodes often emerging immediately after apneas or hypopneas (Ohayon et al., 2001; Hosoya et al., 2014). Of particular clinical relevance, mandibular advancement devices (MAD), which stabilize the airway and reduce respiratory effort, have also been shown to decrease RMMA frequency, suggesting that airway patency and bruxism share common neurophysiological pathways (Hosoya et al., 2014; Lavigne et al., 2007).

These findings motivate a respiratory/airway-protective SB endotype. In this hypothesis, IF the generative model is configured with strong prior preferences for airway patency and a high likelihood of obstructive events, THEN the NESS would include recurrent obstruction–hypoxia–arousal cycles in which RMMA emerges as an active-inference policy to stabilize the mandible, support tongue-base tone, and help restore ventilation. From an FEP standpoint, airway obstruction generates a persistent mismatch between the predicted state of adequate airflow and sensory evidence of reduced ventilation; SB is then interpreted as one of several motor strategies that may contribute to airway reopening and reduction of prediction error, even though this mechanism is primarily inferred from the effects of airway-stabilizing interventions (Hosoya et al., 2014; Lavigne et al., 2007; González-González et al., 2023). Within the NESS framework, this endotype would be associated with craniofacial vulnerability, OSA-like autonomic signatures, and protrusive/lateral wear patterns compatible with airway-supportive mandibular trajectories (Hosoya et al., 2014; Martynowicz et al., 2025).

#### 3.1.4 Dopaminergic/basal ganglia endotypes: other disorders and sleep bruxism

Beyond sleep-related breathing disorders, several neurological, psychiatric, pharmacological, and other sleep-related conditions have been implicated in triggering or exacerbating SB. Neurological disorders such as Parkinson’s disease and epilepsy may alter basal ganglia and dopaminergic circuits, predisposing to abnormal oromotor activity during sleep (Lobbezoo and Naeije, 2001). Psychological conditions – particularly anxiety, stress, and depression – are strongly associated with bruxism and may act through stress-induced sympathetic activation and dysregulated arousal pathways (Manfredini and Lobbezoo, 2009; Pavlou, 2024). Pharmacological factors, including selective serotonin reuptake inhibitors (SSRIs) and dopaminergic agents, have also been linked to increased bruxism frequency, reflecting the role of central neurotransmitter systems in regulating orofacial motor control (Winocur et al., 2003; Garrett and Hawley, 2018). In addition, SB has been reported in association with restless legs syndrome, periodic limb movement disorder, and parasomnias, suggesting partially shared mechanisms of arousal-related instability across multiple sleep disorders (Lobbezoo et al., 2018).

Within the NESS framework, these influences can be grouped into at least one dopaminergic/basal ganglia–weighted SB endotype, acknowledging overlap with stress and arousal pathways. Hypothetically, IF basal ganglia and dopaminergic circuitry are dysregulated – whether due to neurodegenerative disease, acquired brain injury, or medication – THEN a dopaminergic/basal ganglia NESS could be instantiated, whose high-probability states include REM-phase RMMA with incomplete atonia, extreme mandibular excursions, and severe tooth wear despite relatively modest overall RMMA indices. This description highlights a mechanistically distinct pathway by which centrally mediated dysregulation may shape SB, and suggests targeted evaluation of dopaminergic function and sleep architecture in such cases.

### 3.2 Premature contacts and occlusal interferences: peripheral modifiers of NESS expression

Premature contacts and occlusal interferences have long been proposed as mechanical triggers for bruxism, but contemporary evidence suggests their role is secondary rather than causative (Uchima Koecklin et al., 2024; Lavigne et al., 2007). Even though SB is recognized as a centrally mediated oromotor activity, premature contacts – particularly on molars – can influence its *expression* by disturbing occlusal stability and activating protective neuromuscular reflexes. When a tooth contacts prematurely, proprioceptive feedback from the periodontal ligaments prompts compensatory mandibular movements, which may manifest as clenching or grinding (Okeson, 2013). Such local interferences are therefore considered peripheral risk modifiers: they do not initiate SB but may exacerbate muscle hyperactivity, increase grinding frequency, and amplify clinical consequences, including abnormal tooth wear and temporomandibular joint strain (Michelotti et al., 2010; Koyano et al., 2008).

Within the NESS framework, premature contacts and occlusal interferences do not constitute primary SB endotypes, but act as modifiers of how a centrally driven NESS is projected in the orofacial domain. IF a centrally mediated SB endotype (e.g., autonomic/arousal, respiratory, or stress/allostatic) is present, THEN premature occlusal contacts shape the local sensory manifold and reflex pathways such that high-probability states within the NESS include amplified tooth-level loading, increased grinding episodes, and greater clinical damage, without altering the deeper generative model that drives RMMA. This helps explain why occlusal adjustment alone rarely abolishes SB, yet may reduce the severity of its dental consequences (Michelotti et al., 2010; Koyano et al., 2008).

### 3.3 Sleep bruxism as a behavior or a condition: NESS and biological cost

Within the contemporary bruxism literature, an important conceptual distinction is drawn between SB as an adaptive behavior and SB as a condition (Lobbezoo et al., 2018; Raphael et al., 2016; Beddis et al., 2018). This distinction fits naturally within the FEP/NESS framework. RMMA can be viewed as adaptive motor responses – forms of active inference – generated to minimize prediction error when autonomic, cortical, or respiratory cues signal instability in the sleep state (Mayer et al., 2016; Shiraishi et al., 2021; de Miranda Diniz et al., 2024; Zhu et al., 2024). The frequency, interval structure, and intensity of RMMA episodes can be related to homeostatic latency, a proposed temporal descriptor of how efficiently the central nervous system restores equilibrium following micro-arousal–related perturbations. In this context, SB reflects a functional, contextually appropriate behavior within the AAM complex, potentially supporting homeostatic regulation of sleep and, in some individuals, airway patency (González-González et al., 2023; Lavigne et al., 2008; de Miranda Diniz et al., 2024; Hosoya et al., 2014).

Within the NESS framework, the “behavior versus condition” distinction corresponds to whether the instantiated steady-state density remains low-cost and adaptive or shifts toward configurations in which high-probability states include significant biological cost. IF RMMA resolves arousal-related prediction error efficiently and the NESS remains concentrated around low-damage orofacial states, THEN SB is best conceptualized as a behavior, even when frequent, provided it does not entail structural damage or pain. IF maladaptive precision weighting, impaired inhibitory control, heightened sympathetic responsivity, and chronic psychosocial stress reshape the generative model so that prolonged, high-intensity jaw muscle activity, pain states, and tissue damage become characteristic of the NESS, THEN SB manifests as a condition with clinically relevant consequences.

Accordingly, the transition from behavior to condition in SB is not defined by episode frequency or intensity alone, but by the emergence of biological cost and clinical sequelae (Manfredini et al., 2022; Beddis et al., 2018). This view is consistent with the prevailing consensus that bruxism represents a centrally mediated motor behavior that becomes a disorder only when it results in harmful outcomes (Manfredini et al., 2022; Lavigne et al., 2008; Beddis et al., 2018). Within the FEP and NESS framework, this transition can be interpreted as a failure of adaptive predictive regulation: recurrent arousal-related perturbations lead the system to form maladaptive priors anticipating instability, reinforcing chronic bruxism and contributing to pathological consequences (Manfredini et al., 2019).

### 3.4 Sleep bruxism phenotype–endotype mapping

From a review of the nosology of SB, several phenotype classifications emerge (Lobbezoo et al., 2018; Lavigne et al., 2021; Verhoeff et al., 2025; Okuhara et al., 2026). Building on these, we outline a composite set of five descriptive phenotypes – normo-pheno, patho-pheno, SB–OSA comorbid, medication-related SB, and anxiety-weighted SB – as a pragmatic way of linking observable patterns to the proposed endotypes. Each phenotype is hypothesized to constrain different inference channels of the generative model and different temporal coupling patterns; sleep stage and RMMA timing, for example, may help discriminate between autonomic/arousal-dominant, respiratory, and stress/allostatic generative models.

These SB phenotypes can be mapped onto the endotype descriptor framework using the same Bayesian inference/Bayesian inversion logic used in our if-then descriptions: observed clinical and polysomnographic patterns are treated as evidence that updates beliefs about the underlying endotype configuration. Within this scheme, it is the pattern of prediction-error channels – and specifically the putative precision-weighting mechanisms – that is proposed to guide a more granular understanding of SB and, in the future, might inform mechanism-oriented intervention strategies. At present, this phenotype–endotype mapping should be regarded as a structured hypothesis to be tested in prospective studies, rather than as a ready-to-use clinical taxonomy.

### 3.5 Summary of testable hypotheses derived from the framework

The endotype and NESS formulations presented above are intended to be empirically testable, not merely descriptive. In particular, the framework leads to the following falsifiable hypotheses:

#### 3.5.1 Polysomnographic patterns

RMMA in the autonomic/arousal endotype will show systematic clustering within a short latency window after cortical micro arousals and sympathetic bursts, predominantly in N1–N2 sleep, compared with normo pheno SB.

In the respiratory/airway protective endotype, RMMA onset will occur with a characteristic temporal relationship to apnea/hypopnea terminations, and this coupling will weaken when airway stabilizing treatments (e.g., MAD) are effective (Kato et al., 2001; Lavigne et al., 2007; Ohayon et al., 2001; Hosoya et al., 2014; González-González et al., 2023).

#### 3.5.2 Autonomic signatures

Autonomic/arousal and stress/allostatic endotypes will exhibit distinct heart rate and heart rate variability trajectories surrounding RMMA, with higher sympathetic surge amplitude and slower autonomic recovery in stress/allostatic cases (Kato et al., 2001; Lavigne et al., 2007; Shiraishi et al., 2021).

Respiratory/airway protective endotypes will show OSA like autonomic patterns around respiratory events, even when overall apnea–hypopnea indices are in the mild–moderate range (Martynowicz et al., 2019; González-González et al., 2023).

#### 3.5.3 EMG temporal characteristics and homeostatic latency

Individuals with predominantly adaptive, behavioral SB (normo pheno) will show shorter homeostatic latency profiles – i.e., shorter inter episode intervals and faster return of EMG and autonomic variables to baseline – than those with patho pheno SB, controlling for age and comorbidities (Lavigne et al., 2001, 2007; Kato et al., 2001).

Elevated allostatic load (e.g., high perceived stress, pain burden) will be associated with lengthened homeostatic latency and greater clustering of high intensity RMMA episodes across the night (Pavlou, 2024; Mancini et al., 2024).

#### 3.5.4 Airway related phenotypes and treatment response

In SB–OSA comorbid phenotypes, effective MAD or CPAP therapy will not only reduce respiratory events but also selectively decrease RMMA episodes temporally linked to apnea/hypopnea, while leaving stress related or dopaminergic related episodes relatively unchanged (Hosoya et al., 2014; Lavigne et al., 2007; Li et al., 2023; Doblado et al., 2025).

Craniofacial morphologies associated with airway vulnerability (e.g., retrognathia, narrow maxilla) will be over represented in respiratory/airway protective endotypes compared with normo pheno SB (González-González et al., 2023; Martynowicz et al., 2019).

#### 3.5.5 Biomarker and endotype profiles

Stress/allostatic endotypes will show higher levels of stress related biomarkers (e.g., cortisol, catecholamines) and higher self-reported stress and maladaptive coping scores than autonomic/arousal or respiratory/airway protective endotypes (Karakoulaki et al., 2015; Kaya et al., 2025; Walczyńska-Dragon et al., 2025; Saczuk et al., 2019).

Dopaminergic/basal ganglia-weighted endotypes will exhibit a higher prevalence of Parkinson’s disease, epilepsy, dopaminergic/serotonergic medication use, or associated movement disorders than other SB presentations (Lobbezoo and Naeije, 2001; Winocur et al., 2003; Garrett and Hawley, 2018; Lobbezoo et al., 2018).

Collectively, these hypotheses specify observable PSG, autonomic, EMG, craniofacial, and biomarker patterns that can be examined in future prospective cohorts to evaluate whether the proposed FEP informed endotypes and the construct of homeostatic latency capture clinically meaningful variation in SB.

## 4 Clinical and computational implications of an FEP-informed ontology

Reframing SB through the FEP allows it to be conceptualized not merely as a behavioral or oromotor symptom presentation, but as an active, inferential, context-dependent process, consistent with the framework developed in Parts 2 and 3. Within this view, SB episodes are interpreted as policies recruited when sensory input violates prior predictions under a given generative model, with the organism attempting to minimize expected VFE and reduce uncertainty about bodily states during sleep (Ororbia and Friston, 2023; Parr et al., 2020). This interpretation remains theoretical and is intended to generate hypotheses, not to assert empirically established mechanisms (Da Costa et al., 2022).

An FEP-informed ontological description of SB endotypes that captures relevant sensory and other raw data, compresses these into tractable features, and links them to specific phenotypes through ontology mapping can render such hypotheses computationally explicit (Da Costa et al., 2022; HL7 International, 2018; National Center for Biotechnology Information [NCBI], 2024). Within this ontology, each bruxing event with its temporal coupling signature can be modeled as a transient excursion from a homeostatic attractor, with homeostatic latency marking its asymptotic boundary and reflecting the system’s conceptual return to expected physiological states, while successive events define a homeorhetic trajectory of SB expression and potential allostatic load (Ororbia and Friston, 2023). At present, homeostatic latency remains a theoretical construct; specific operational definitions, measurement protocols based on PSG/HSAT and masseter EMG, and reliability studies are required before it can be treated as a clinical parameter.

From a clinical standpoint, the framework suggests a move from purely frequency-based grading of SB toward more mechanism-informed phenotyping. The proposed autonomic–arousal, stress/allostatic, respiratory-airway protective, and neuromodulatory/basal-ganglia endotypes provide a vocabulary for organizing polysomnographic, autonomic, craniofacial and psychosocial observations, but should be regarded as working hypotheses rather than validated diagnostic categories (Lobbezoo et al., 2018; Svensson and Lavigne, 2020; Lavigne et al., 2021; Verhoeff et al., 2025; Okuhara et al., 2026; Meira e Cruz, 2026). This aligns with contemporary consensus efforts and may support more precise, structured grading of SB presentations when combined with established definitions and indices (e.g., ICD-11, BEWE, STAB) and instrumentally generated data such as digital STL difference files, PSG/HSAT, intraoral sensor data, and photogrammetry (Manfredini et al., 2024; Souza et al., 2020). However, endotype-based treatment algorithms or prognostic tools are not yet justified and should remain future research targets.

Within this perspective, conventional stand-alone SB interventions can be constructively re-examined. Oral appliances, for example, can be conceptualized as inputs that modulate bruxing events and their trajectories within the generative model, while adjunctive interventions such as cognitive behavioral therapy, botulinum toxin injections, and other modalities may be combined to manage downstream allostatic load (Manfredini et al., 2024). Homeostatic latency and homeorhetic trajectory concepts could eventually provide more mechanistically interpretable outcome measures for such multimodal strategies, but this will require empirical validation and should, at present, be considered hypothesis-generating (Fisher et al., 2018; Edmund Tay Mai Hiong Endowed, 2018).

On a computational level, the FEP-informed ontology points toward, but does not yet realize, a domain-specific knowledge graph (KG) for SB. A KG could formally represent entities (e.g., SB phenotypes and proposed endotypes, craniofacial configurations, autonomic signatures, SB indices), attributes, and relationships (e.g., precedes, modulates, co-occurs with), preserving relational structure in a way that conventional tabular databases cannot natively capture (Da Costa et al., 2022; Wang et al., 2025; Wu et al., 2025). Such a KG might eventually support graph-based querying of temporal coupling patterns, integration of multimodal data streams, and mechanistically informed hypothesis testing about state transitions across homeostatic, homeorhetic, and allostatic regimes (Ororbia and Friston, 2023).

In principle, this ontological-KG architecture could later be linked to machine-learning methods and, ultimately, to electronic health records and clinical decision support systems (HL7 International, 2018; Da Costa et al., 2022; Wang et al., 2025). However, no such KG, EHR integration, or decision support tool currently exists for SB, and the present article does not propose or validate an operational system. References to precision dentistry, closed-loop decision processes, or AI-driven stratification should therefore be interpreted as outlining a potential long-term research trajectory contingent on multiple intermediate steps – formalizing the ontology, validating endotypes and temporal metrics such as homeostatic latency, and demonstrating added value beyond existing models – rather than as immediate clinical applications.

## 5 Conclusion and future directions

Viewed through the lens of the FEP, SB can be interpreted as a context-sensitive motor response that may be recruited to resolve transient prediction error and support physiological stability during arousal-related perturbations in sleep. In this perspective, stress, arousals, respiratory events, and oromotor output are integrated within a broader autonomic–arousal–motor complex, so that SB is framed not merely as a peripheral parafunction, but as a potential active-inference policy enacted under generative models of sleep and airway stability. This interpretation is theoretical and intended to organize existing findings and generate testable predictions, rather than to claim a fully established mechanism.

Within this framework, we have outlined a candidate mechanistic account of SB grounded in the FEP, with active inference providing the core process by which neural systems are proposed to minimize VFE. The construct of homeostatic latency was introduced as a conceptual descriptor of the temporal window over which RMMA frequency, inter-episode intervals, and contraction intensity reflect the efficiency of returning toward quasi-stable states after arousal-related perturbations in nested craniofacial Markov-blanket systems. This allows SB tendencies to be positioned along a continuum from predominantly adaptive, low-cost behavior to more persistent, high-cost endotype expressions associated with increased allostatic load. At present, homeostatic latency remains a hypothesis-generating construct that requires operationalization and empirical validation before clinical use.

The central conjecture advanced here is that deleterious SB phenotype–endotype dynamics may reflect failures or biases in adaptive Bayesian inference within the craniofacial and autonomic domains. In this view, specific endotypes (e.g., autonomic/arousal-dominant, stress/allostatic, respiratory/airway-protective, dopaminergic/basal-ganglia – related) correspond to different parametric configurations of the underlying generative model and NESS, which in turn give rise to characteristic patterns of RMMA timing, autonomic signatures, craniofacial loading, and clinical sequelae. Homeostatic latency, as defined in Section 2.3, is proposed as a temporal descriptor of neuromuscular regulatory trajectories that could, in principle, help quantify risk, track homeorhetic progression, and approximate cumulative allostatic load – pending rigorous methodological development.

Future work should first focus on empirical testing of the hypotheses derived from this framework, rather than on immediate clinical deployment. Prospective studies integrating polysomnography, EMG, autonomic measures, craniofacial phenotyping, and stress-related biomarkers are needed to evaluate whether the proposed endotypes and homeostatic latency profiles capture clinically meaningful variation in SB and its comorbid presentations. In parallel, careful psychometric and signal-processing work will be required to define, compute, and validate temporal metrics such as homeostatic latency across nights and recording systems.

On a longer time horizon, the FEP-informed ontology and NESS formulation presented here could contribute to ontology- and knowledge-graph – based representations of SB, supporting more systematic integration of multimodal data (e.g., PSG/HSAT metrics, digital STL datasets, intraoral sensors, and clinical records). Such computational structures may eventually facilitate more refined phenotyping, endotype inference, and hypothesis-driven machine-learning analyses, but their clinical value will depend on stepwise validation and demonstration of added explanatory or predictive power over existing consensus-based approaches. In this sense, the present work is best understood as a conceptual bridge between current bruxism research and potential future clinical and computational tools, aiming to stimulate empirical studies that can confirm, refine, or falsify the proposed FEP-based account of SB.

Finally, it is important to acknowledge that the FEP and Markov blanket formalisms remain topics of active debate, including technical critiques of some formulations and discussions about their scope and applicability to concrete biological systems (e.g., Biehl et al., 2021; related commentaries). In light of these ongoing discussions, the present framework should be read as one possible, explicitly theoretical lens for organizing current data and generating empirically testable predictions about SB, rather than as a definitive or exclusive explanatory model.

## Data Availability

The original contributions presented in the study are included in the article/supplementary material, further inquiries can be directed to the corresponding author.
